# Pedigree data indicate rapid inbreeding and loss of genetic diversity within populations of native, traditional dog breeds of conservation concern

**DOI:** 10.1371/journal.pone.0202849

**Published:** 2018-09-12

**Authors:** Mija Jansson, Linda Laikre

**Affiliations:** Department of Zoology, Division of Population Genetics, Stockholm University, Stockholm, Sweden; Senckenberg am Meer Deutsches Zentrum fur Marine Biodiversitatsforschung, GERMANY

## Abstract

Increasing concern is directed towards genetic diversity of domestic animal populations because strong selective breeding can rapidly deplete genetic diversity of socio-economically valuable animals. International conservation policy identifies minimizing genetic erosion of domesticated animals as a key biodiversity target. We used breeding records to assess potential indications of inbreeding and loss of founder allelic diversity in 12 native Swedish dog breeds, traditional to the country, ten of which have been identified by authorities as of conservation concern. The pedigrees dated back to the mid-1900, comprising 5–11 generations and 350–66,500 individuals per pedigree. We assessed rates of inbreeding and potential indications of loss of genetic variation by measuring inbreeding coefficients and remaining number of founder alleles at five points in time during 1980–2012. We found average inbreeding coefficients among breeds to double–from an average of 0.03 in 1980 to 0.07 in 2012 –in spite of the majority of breeds being numerically large with pedigrees comprising thousands of individuals indicating that such rapid increase of inbreeding should have been possible to avoid. We also found indications of extensive loss of intra-breed variation; on average 70 percent of founder alleles are lost during 1980–2012. Explicit conservation goals for these breeds were not reflected in pedigree based conservation genetic measures; breeding needs to focus more on retaining genetic variation, and supplementary genomic analyses of these breeds are highly warranted in order to find out the extent to which the trends indicated here are reflected over the genomes of these breeds.

## Introduction

Domestic animals are often bred under strong selection that focuses on a few specific traits, and this type of breeding is considered to result in loss of genetic biodiversity both in the form of entire breeds–typically old, traditional, local ones–and in variation within breeds [1]. Increasing focus is directed towards genetic diversity of domestic animal populations both scientifically [2, 3, 4] and politically [5]. The Strategic Plan for Biodiversity 2011–2020 adopted by the 196 parties to the United Nations Framework Convention on Biological Diversity (CBD; http://www.cbd.int/sp) stresses that by 2020 the genetic diversity of domesticated animals and other species of socio-economic and cultural value should be maintained. Similarly, strategies for safeguarding the genetic diversity of such species should be developed and implemented over this time period (Aichi Target 13; http://www.cbd.int/sp/targets).

Food producing animals have been the main targets for conservation actions [5], but horses, dogs, cats, rabbits that in many countries are primarily used for other purposes are becoming increasingly recognized as of conservation value [6, 7]. Countries are reviewing their national domestic animal breeds [8], and in Sweden the Swedish National Board of Agriculture has identified 63 such breeds historically originating in Sweden, and has declared them of national conservation concern. These Swedish national breeds include 8 cattle, 1 pig, 11 sheep, 4 goat, 2 goose, 4 duck, 4 horse, 12 chicken, 2 rabbit, 2 cat, and 10 dog breeds [9].

The domestic dog renders increased socio-economic importance due to its many roles in modern society. Dogs of different breeds are used for human therapy, sports activities, herding, hunting, medical research, identifying biological material, companionship, as well as in police, customs, military, rescue, and security work, [10, 11, 12, 13].

At the same time, recent research indicates that within breed genetic diversity is rapidly depleted [14, 15, 16, 17] and this can hamper long term maintenance of separate breeds. This is a concern not the least for old, traditional breeds that represent a biological and cultural heritage that is becoming increasingly recognized [18, 19, 20, 21].

**Fig 1:**
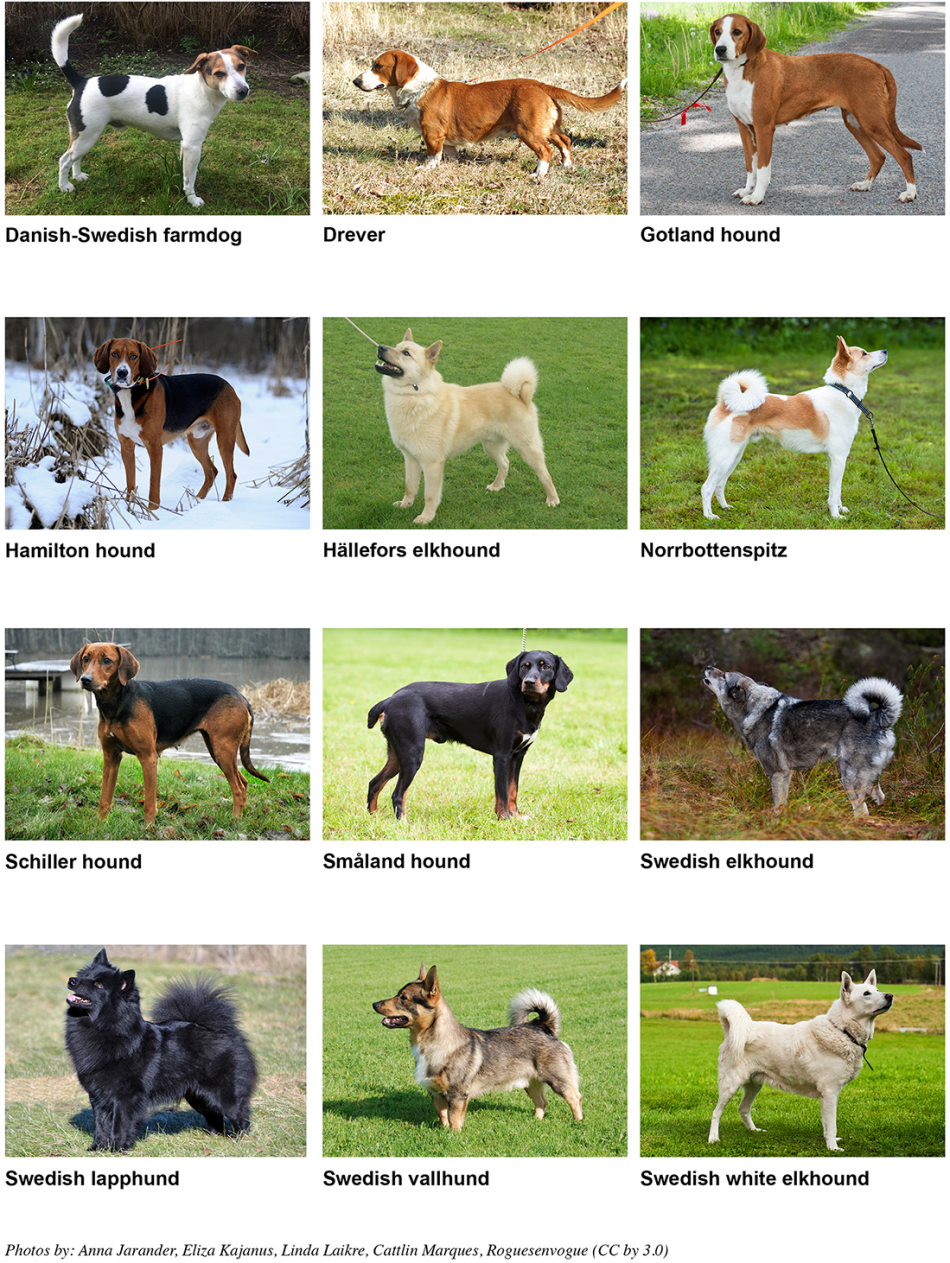
The 12 national Swedish traditional dog breeds that are monitored based on pedigree data in this study. All of these breeds except Hällefors elkhound and Swedish white elkhound have been identified as of conservation concern by national authorities [9, 24].

In this study, we used extensive pedigree data provided by the Swedish Kennel Club comprising the past few decades to assess rates of inbreeding and loss of intra-breed variation measured in terms of number of founder alleles [22, 23] in 12 dog breeds originating in Sweden (Fig 1). Of these breeds ten have been identified as of national conservation concern; four have been classified as endangered-maintained, and six as critical-maintained [24] using the FAO classification system for domestic animal populations [8]. We were particularly interested in monitoring whether the increased conservation genetic concern is reflected in pedigree measures of diversity since pedigree data from breeding records constitutes the basis for dog breed clubs and dog breeders when planning breeding.

## Materials and methods

Pedigree data for the 12 Swedish domestic dog breed populations used in this study was obtained from the Swedish Kennel Club (SKC; www.skk.se; a kennel club is a nationwide organization which works and cares for purebred dogs in a country). SKC was founded in 1889 and their computerized studbook database was created in 1975–76. The database comprises pedigrees of almost all dog breeds kept in Sweden and approximately 90 percent of pedigree dogs in Sweden are included in these records (SKC, pers. comm.).

Ten of the 12 breeds have been classified as of Swedish national conservation concern [9, 24] (Table 1). The data we used consisted of the full pedigree information from the start of the pedigrees and up until December 31, 2012. The start of the pedigrees varies between breeds (Table 1), with the birth of the first dogs included in the pedigrees varying from the 1940s (Drever, Hamilton hound, Schiller hound, Småland hound, and Swedish elkhound) to the 1990s (Swedish white elkhound). We obtained strategic breeding plans for each of the 12 breeds from the Swedish Kennel Club’s website (www.skk.se) and reviewed these plans to find out whether 1) retention of genetic variation is an explicit breeding goal for the separate breeds, 2) whether disease control programs for genetic diseases/disorders are considered for the breed. The strategic breeding plans are produced by breeding clubs associated to the SKC, and SKC has requested breeding clubs to produce and continuously revise such plans for each breed since around year 2000. We downloaded and used the plans that applied in 2012 from the SKC website (www.skk.se; the plans are in Swedish).

**Table 1:** Basic information on the 12 native Swedish dog breeds assessed in this study.

Breed	Traditional use	Swedish national conservation concern^1^	Goal to maintain genetic variation^2^	Conservation classification^3^	Disease control programs^4^	Year of SKC recognition^5^	Start of pedigree^6^
Danish-Swedish farmdog	Vermin catcher and watch dog	Yes	Yes	Endangered-maintained	Hip dysplasia	1987	1980s
Drever	Roe buck and deer hunting	Yes	Yes	Endangered-maintained	None	1913	1940s
Gotland hound	Hare and fox hunting	Yes	Yes	Critical-maintained	None	1990	1980s
Hamilton hound	Hare and fox hunting	Yes	Yes	Endangered-maintained	None	1886	1940s
Hällefors elkhound	Elk hunting	Not yet	Yes	Not assessed	None	2000	1950s
Norrbottenspitz	Versatile hunting	Yes	Yes	Critical-maintained	Progressive retinal atrophy	1967	1960s
Schiller hound	Fox and hare hunting	Yes	Yes	Critical-maintained	None	1907	1940s
Småland hound	All round hunting	Yes	Yes	Critical-maintained	None	1921	1940s
Swedish elkhound	Elk and carnivore hunting	Yes	Yes	Endangered-maintained	Hip dysplasia	1946	1940s
Swedish lapphund	Herding reindeer, hunting	Yes	Yes	Critical-maintained	Hip dysplasia, progressive retinal atrophy	1893	1950s
Swedish vallhund	Herding cattle, watch dog	Yes	Yes	Critical-maintained	None	1943	1950s
Swedish white elkhound	Elk hunting	Not yet	Yes	Not assessed	Hip dysplasia	1993	1990s

^1^Classification by Swedish Board of Agriculture [9].

^2^Refers to whether the breeding strategy as of 2012 stated that a main goal is retention of genetic diversity.

^3^Performed by the Swedish Board of Agriculture [24] using the FAO classification system [8].

^4^Refer to whether a strategy aimed at reducing specific hereditary/familiar disorders is implemented for the breed (at 2012).

^5^ Year of SKC officially recognized the particular breed as a pure breed under the SKC and began to keep studbook data.

^6^The earliest decade in which individuals of the pedigrees used in this study were born.

To monitor potential temporal trends we analyzed levels of inbreeding and loss of founder allele variation (see below) at five points in time including dogs alive on December 31 in the years of 1980, 1990, 2000, 2006, 2012, respectively. To determine the number of live dogs at the five points in time we had to make assumptions about the longevity of dogs. This is because the SKC studbooks do not include information on date of death of separate individuals. We assumed that each dog lives for 12 years after its date of birth. For example, a dog born on January 1, 1999 was assigned a date of death on January 1, 2011. Thus, our analysis of dogs alive in e.g. December 31, 2000 includes animals born between January 1, 1989 and December 31, 2000. Using this definition the number of living individuals as of December 31, 2012 ranged from 149 (Gotland hound) to 17,483 (Swedish elkhound).

The SKC pedigrees include individuals of other countries if such individuals have descendants in the Swedish populations. Thus, when a dog is imported, or when a Swedish dog is mated to a dog abroad, pedigree information a few generations back that include dogs with foreign registration numbers are included in the Swedish studbooks. We have always excluded such dogs among those classified as alive since these dogs do not exist within the Swedish populations. We only included dogs with a Swedish registration number as being alive and dogs with foreign registration numbers that should be alive according to the maximum age of 12 years criterion have been marked as dead, while they do not belong to the Swedish population. A complete date of birth is required for the applied pedigree software (see below) and we therefore assigned suitable dates of birth to individuals lacking this data (approximately 5 percent of the individuals) based on existing information (i.e. birth dates of parents and/or offspring in the studbook). We used the mPed software [25] to perform such modifications of birth and death dates. For 0.2 percent of the individuals lacking birth information no guiding data were available and thus no approximate birth date could be assigned.

### Pedigree analysis

Pedigree analysis was used to assess inbreeding and loss of genetic variation in relation to the population founders over the time period monitored. A founder is an individual unrelated to all other individuals in the pedigree except for its descendants; at any random locus, it carries two hypothetical, unique founder alleles. The coefficient of inbreeding (*F*) and mean kinship (*MK*) quantify probabilities of identity by descent (IBD) of such alleles, within and between individuals, respectively [23]. Genetic variation is measured as the number of remaining founder alleles and the number of founder genome equivalents (*fge)* [23]. *fge* is the number of equally contributing founders from which no random loss of alleles has occurred that represent the same amount of variation as that retained in the living population. *fge* should be compared to the true number of founders; difference results from loss of founder alleles and uneven genetic contribution of separate founders to presently living individuals [23].

We used the Population Management x software (PMx) [25] to obtain the quantities assessed using the default value of 1,000 iterations for gene drop simulations. The program mPed [26] was used to convert the studbook data obtained from the SKC to PMx input format and to “strip” pedigrees to sizes possible to handle by PMx. This software can only deal with pedigrees comprising around 20,000 individuals depending on the complexity of the pedigree. “Stripping” implies removing individuals from the pedigree which do not contribute information on individuals alive, i.e., dead individuals that do not have any living descendants. For pedigrees of some breeds at particular points in time the pedigrees were too large even after stripping. Those cases could not be analyzed and were excluded from this study; Drever (1990, 2000, and 2006), Hamilton hound (1990), and Swedish elkhound (2006 and 2012). We also used PMx to describe inbreeding rates in terms of effective population size (*N*_e_) defined in two ways: 1) as per generation average effective population size over the analysis time span, and 2) as “current effective population size” of the living population based on the number of living males and females that have produced offspring using the equation *N*_e_ = 4*N*_m_*N*_f_/(*N*_m_+*N*_f_) where *N*_*m*_ = number of live males that have reproduced and *N*_*f*_ = number of living females that have reproduced [25].

To evaluate potential differences in the parameters assessed over time within breeds we used analyses of variance tests (ANOVAs) performed with MS Excel and R 2.12.2. The latter software was also used for Kruskal-Wallis tests. Two-factor ANOVAs were performed in MS Excel. Because of the unbalanced data sets with large differences in number of observations per breed and point in time, we used two-factor ANOVAs without replication. MS Excel was also used for t-tests and linear regression analyses.

## Results

The size, depth (number of generations), and number of founders of the full pedigrees as of December 31, 2012 varies greatly for separate breeds (Table 2). The numerically smallest breed is the Gotland hound whose full pedigree includes only 350 individuals descending from 12 founders, whereas the Drever is the largest breed with over 66,000 individuals in the full pedigree descending from 316 founders. The number of generations over the study period varies from 4.6 generations in the Swedish white elkhound to 11.4 for the Swedish vallhund. For three breeds very few individuals are defined as alive at our earliest time point of analysis in December 31, 1980; the Gotland hound (2 individuals), the Swedish white elkhound (5 individuals), and the Danish-Swedish farmdog (23 individuals). We have performed analyses both including and excluding these data. Retention of genetic variation is an explicit breeding goal for all of the 12 breeds according to the breed specific strategic breeding plans. Disease control programs for genetic diseases/disorders were in operation for five breeds in 2012 (Table 1).

**Table 2:** Summary statistics for the 12 Swedish dog breeds of this study over five points in time (date of assessment was December 31 for all five years).

Breed	Year of assessment	*N* in pedigree^1^	No of founders^2^	*N* alive^3^	*F* mean^4^	*F* range^5^		*MK* mean^6^	*MK* range^7^	Proportion remaining founder alleles^8^	*Fge*^9^	*N*_*e*_^10^	*G*^11^	Current *N*_*e*_^1^^2^	Current *N*_*e*_*/N*^13^
Danish-Swedish farmdog	1980	24	20	23	0.000	0.000–0.000		0.034	0.022–0.065	0.913	14.69	21.3	0.4	3.2	0.14
	1990	441	57	413	0.016	0.000–0.313		0.046	0.001–0.093	0.863	10.81	24.2	1.9	71.6	0.17
	2000	2161	74	1793	0.042	0.000–0.340		0.056	0.000–0.092	0.62	8.94	35.7	3.6	236	0.13
	2006	4588	110	3416	0.042	0.000–0.340		0.05	0.000–0.078	0.548	10.05	51.3	4.7	502.1	0.15
	2012	8762	124	6272	0.041	0.000–0.295		0.044	0.000–0.067	0.493	11.32	71.4	5.9	871.1	0.14
Drever^14^	1980	17003	620	15700	0.039	0.000–0.325		0.027	0.000–0.059	0.596	18.28	93.2	5	3084.3	0.20
	2012	66458	316	10010	0.073	0.000–0.296		0.067	0.005–0.096	0.141	7.5	79.4	10.7	750.6	0.07
Gotland hound	1980	6	4	2	0.000	0.000–0.000		0.25	0.250–0.250	0.5	2	3.5	1	0	0.00
	1990	49	15	37	0.000	0.000–0.000		0.072	0.014–0.110	0.77	6.93	18.2	1.5	3.3	0.09
	2000	201	14	173	0.06	0.000–0.156		0.099	0.028–0.143	0.741	5.08	22	2.9	27	0.16
	2006	256	12	125	0.106	0.008–0.289		0.132	0.068–0.181	0.583	3.8	19.1	3.7	11.7	0.09
	2012	350	12	149	0.103	0.008–0.289		0.151	0.071–0.185	0.484	3.31	19.5	4.7	24	0.16
Hamilton hound^14^	1980	14320	532	13100	0.038	0.000–0.302		0.028	0.000–0.056	0.638	17.98	85.5	4.7	2488	0.19
	2000	41881	317	13456	0.057	0.000–0.310		0.056	0.002–0.085	0.215	9	68.5	7.6	892.7	0.07
	2006	45180	291	8097	0.061	0.012–0.310		0.063	0.019–0.083	0.159	7.98	68.7	8.6	509.6	0.06
	2012	47092	268	5121	0.06	0.016–0.219		0.066	0.027–0.081	0.125	7.6	72.4	9.6	311.6	0.06
Hällefors elkhound	1980	95	53	79	0.011	0.000–0.125		0.023	0.006–0.064	0.851	21.57	37.1	1	30.2	0.38
	1990	293	67	221	0.039	0.000–0.250		0.052	0.002–0.104	0.673	9.58	31.7	2.9	79.5	0.36
	2000	809	65	575	0.074	0.000–0.237		0.086	0.001–0.136	0.47	5.83	31.5	5.1	97.3	0.17
	2006	1171	59	691	0.087	0.000–0.237		0.102	0.001–0.154	0.444	4.88	31.6	6.2	94.5	0.14
	2012	1483	55	670	0.085	0.000–0.193		0.106	0.001–0.144	0.376	4.72	34.7	7.1	90.9	0.14
Norrbotten-spitz	1980	2001	76	1903	0.039	0.000–0.254		0.041	0.000–0.084	0.708	12.13	41.2	2.9	367.6	0.19
	1990	5211	60	3829	0.05	0.000–0.320		0.046	0.003–0.075	0.581	10.78	53.2	4.1	407.9	0.11
	2000	6750	67	1882	0.048	0.000–0.307		0.053	0.001–0.080	0.383	9.39	61.1	5.7	181.8	0.10
	2006	7638	94	1549	0.046	0.000–0.292		0.06	0.001–0.081	0.303	8.4	61.5	6.9	186.4	0.12
	2012	8526	116	1626	0.049	0.000–0.232		0.062	0.000–0.085	0.269	8.07	66.7	7.9	222.3	0.14
Schiller hound	1980	5211	183	4778	0.044	0.000–0.272		0.047	0.000–0.078	0.657	10.66	48.3	4.4	866.6	0.18
	1990	11246	124	6126	0.055	0.000–0.289		0.058	0.000–0.080	0.404	8.66	51.4	5.7	425.5	0.07
	2000	10067	111	2724	0.062	0.000–0.308		0.073	0.028–0.095	0.241	6.83	51.3	7.3	188.1	0.07
	2006	12906	101	1901	0.067	0.022–0.297		0.077	0.049–0.101	0.196	6.47	55.7	8.4	177.1	0.09
	2012	13906	106	1699	0.068	0.000–0.290		0.079	0.042–0.094	0.194	6.31	59.7	9.3	161.2	0.09
Småland hound	1980	2738	143	2135	0.036	0.000–0.318		0.032	0.000–0.062	0.613	15.55	74.2	4.3	546.9	0.26
	1990	4966	127	3131	0.05	0.000–0.318		0.05	0.007–0.082	0.373	10.06	63.1	5.9	317.9	0.10
	2000	6259	112	1676	0.051	0.013–0.302		0.06	0.035–0.077	0.232	8.32	65.5	7.5	141.9	0.08
	2006	6138	108	1176	0.055	0.000–0.302		0.07	0.031–0.094	0.186	7.17	63.7	8.6	113.8	0.10
	2012	7239	109	977	0.063	0.000–0.184		0.08	0.030–0.100	0.168	6.29	61.6	9.6	93.1	0.10
Swedish elkhound^14^	1980	8113	280	7258	0.067	0.000–0.540		0.052	0.000–0.127	0.616	9.64	48.3	4.9	1525.1	0.21
	1990	20397	223	14213	0.076	0.000–0.540		0.066	0.000–0.134	0.349	7.55	49.7	6.6	1353.3	0.10
	2000	33541	228	17483	0.078	0.003–0.471		0.076	0.015–0.129	0.239	6.55	54.3	8.4	1690.7	0.10
Swedish lapphund	1980	2143	65	1979	0.056	0.000–0.268		0.052	0.000–0.086	0.634	9.65	48.9	4.4	360.7	0.18
	1990	2143	55	3032	0.089	0.004–0.315		0.067	0.023–0.098	0.44	7.42	49.7	6	332.8	0.11
	2000	6187	51	1896	0.09	0.008–0.337		0.077	0.033–0.115	0.383	6.52	55.4	7.7	197.5	0.10
	2006	6961	54	1500	0.083	0.000–0.337		0.082	0.031–0.120	0.31	6.09	57.7	8.8	202.7	0.14
	2012	7482	64	1213	0.081	0.000–0.283		0.084	0.032–0.121	0.267	5.93	58.4	9.8	166.1	0.14
Swedish vallhund	1980	1573	68	1410	0.061	0.000–0.230		0.057	0.000–0.106	0.675	8.83	50.4	5.1	321.6	0.23
	1990	3904	58	2724	0.077	0.000–0.313		0.075	0.020–0.110	0.386	6.64	50.8	7	294.1	0.11
	2000	5794	73	2234	0.091	0.000–0.333		0.09	0.004–0.118	0.323	5.57	52.4	9.1	286.2	0.13
	2006	7199	85	2342	0.09	0.000–0.333		0.091	0.012–0.112	0.237	5.47	57.9	10.4	337.4	0.14
	2012	8490	98	2509	0.089	0.000–0.254		0.092	0.039–0.110	0.215	5.44	62.4	11.4	313.7	0.13
Swedish white elkhound	1980	18	9	5	0.000	0.000–0.000		0.11	0.100–0.125	0.528	4.55	8.7	1	0	0.00
	1990	290	64	219	0.000	0.000–0.000		0.021	0.002–0.051	0.806	23.91	48.5	1.3	16.9	0.08
	2000	1084	81	867	0.003	0.000–0.063		0.025	0.001–0.044	0.813	19.89	62.8	2.4	119.2	0.14
	2006	1548	62	869	0.01	0.000–0.070		0.037	0.001–0.053	0.533	13.64	60.2	3.5	94.8	0.11
	2012	2051	58	915	0.018	0.000–0.070		0.044	0.001–0.063	0.468	11.49	63.9	4.6	101.5	0.11

^1^Total number of individuals in the pedigree.

^2^The number of presumably unrelated individuals that contribute genes to the living individuals.

^3^Number of living animals.

^4^Mean inbreeding coefficient among live animals.

^5^The range of inbreeding coefficients among living individuals.

^6^Average mean kinship values for living individuals.

^7^Range of MKs among living dogs.

^8^The proportion of the initial number of founder alleles (defined as 2 x the number of founders) that is retained in one or more copies among living dogs.

^9^Number of founder genome equivalents represented in living animals.

^10^Average genetically effective population size over generations.

^11^Number of generations represented in the pedigree.

^12^Estimated as *N*_*e*_ = 4*N*_*m*_*N*_*f*_/(*N*_*m*_+*N*_*f*_), where *N*_*m*_ = number of live males that have reproduced and *N*_*f*_ = number of living females that have reproduced.

^13^Current *N*_*e*_ in relation to current census size (*N*).

^14^The pedigrees for these breeds were too complex to be analyzed at 1–3 points in time, and data for those dates are missing.

### Inbreeding

**Fig 2:**
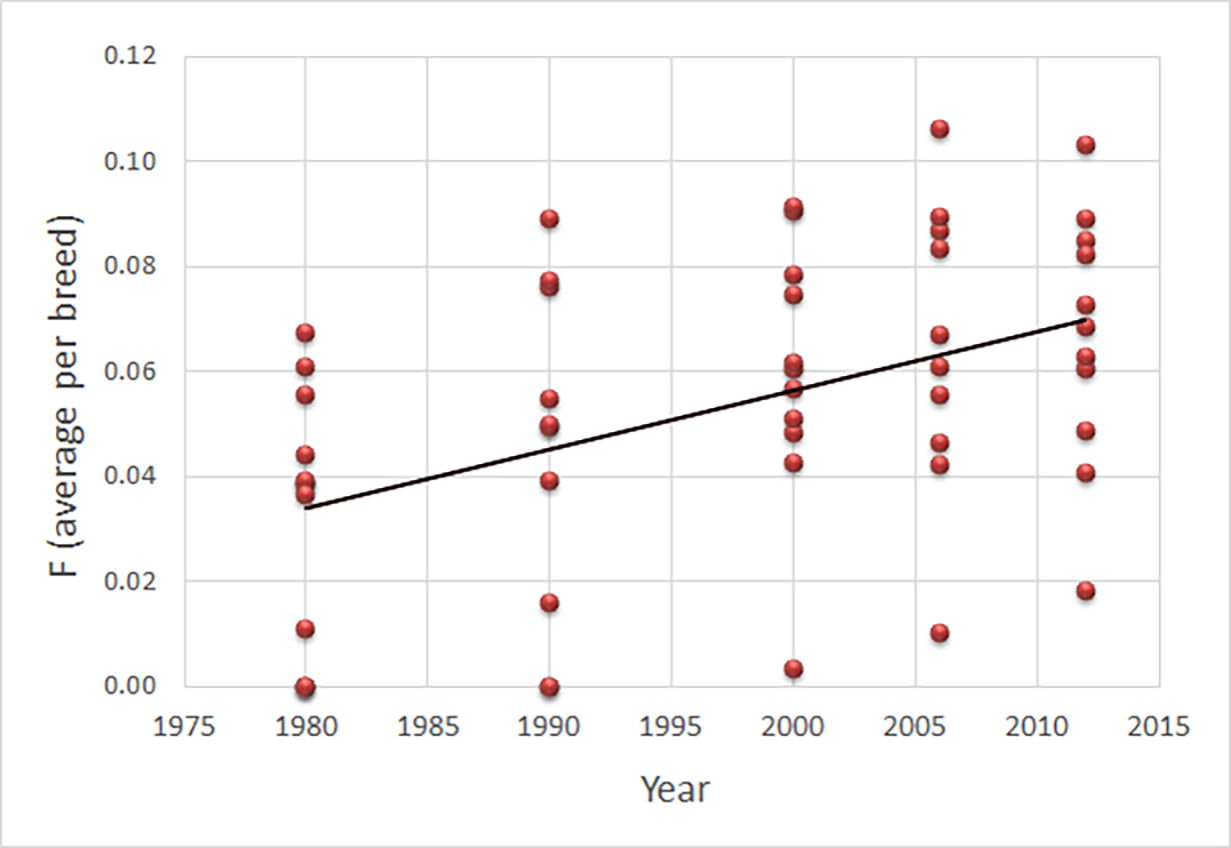
Average per breed inbreeding level in 12 Swedish traditional dog breeds measured from pedigree data at five points in time during 1980–2012. Linear regression gives *b* = 0.001, *r*^2^ = 0.21, P<0.001 and the regression line is shown.

The recent rates of inbreeding that we were able to monitor with the existing pedigree data is relatively extensive and the average coefficient of inbreeding (*F*) over the studied breeds increase over the five points in time from 0.033 in 1980 to 0.066 in 2012, exceeding the level of first cousin mating (*F*>0.0625) in both 2006 and 2012 (cf. Fig 2; Table 2; S1 Fig). There is no correlation between average *F* and population size measured either as the size of the full pedigree or as the number of living dogs (coefficients of correlation, *r*, range from 0.00 to 0.53 and 0.15–0.60, respectively, with 0.07<P<1.00). Similarly, there is no correlation between *F* and the number of founders at any of the points in time (0.03<*r*<0.45; 0.19<P<0.93), suggesting that, contrary to what might be expected, the number of founders or the population size does not explain inbreeding levels. For the Gotland hound, census numbers have been consistently low over the study period with the number of animals regarded as alive never exceeding 180. Similarly, the number of founders is very low for this particular breed (maximum of 15 in 1990) and average levels of inbreeding is also highest in this breed with average *F* = 0.10 in 2012. Breeds like the Swedish lapphund and the Swedish vallhund, however, reach almost as high average *F—*0.09—in spite of pedigree sizes of several thousand individuals, census sizes of well over 1000, and over 50 founders. Similarly, the Swedish elkhound and the Drever have pedigrees comprising over 50,000 dogs, with over 10,000 defined as alive in 2012 but average *F* is over 0.07.

Mean kinship values (*MK*; Table 1) at particular points in time show the average relatedness among living individuals [23]. *MK* is used in conservation breeding to choose animals for breeding; by prioritizing individuals with low *MK* inbreeding and loss of founder genetic variation is minimized. Average and range of *MK*s among the Swedish dog breeds show that such prioritization has typically not been carried out for these breeds. In some cases average *MK* at one point in time is lower than average *F* for the next time point. For instance, average *F* for the Swedish lapphund is consistently larger than the average *MK* for the prior time point (Table 2) indicating that increase of inbreeding is unnecessary high in this breed. In contrast, for the Gotland hound, the Swedish white elkhound, the Norrbottenspitz, and the Danish-Swedish farmdog average *F* is below the *MK* of the preceding time step indicating that dogs chosen for breeding have had lower *MK* than the average among living dogs.

### Differences in inbreeding among breeds and over time

Inbreeding increases over time as expressed by a significant linear relationship between time and average inbreeding coefficient per breed (*b* = 0.001, *r* = 0.46, P<0.001; Fig 2). There are differences among breeds; a two-factor ANOVA without replication yields significance for both breed differences (between breeds *F*_8,32_ = 9.60, P<<0.001) and point in time (between time point *F*_4,32_ = 8.77, P<<0.001). This analysis included all five points in time and only the nine breeds for which results from all time points are available (Table 2). Removing 1980 (when the pedigrees for three of the breeds have just started; Table 1) still gives significant results for both among breeds (*F*_8,24_ = 9.40, P<<0.001) and among points in time (*F*_3,24_ = 4.33, P<0.05).

Breed differences persist also over the final part of our time series, 2000–2012 (*F*_8,16_ = 26.88, P<<0.001), but significant differences between the three time points (2000, 2006 and 2012) are lacking (*F*_2,16_ = 2.24, P = 0.14). The Hamilton hound lacks results from the full five point time series (Table 2) but has results from 2000, 2006, and 2012, and could thus be included for these time points. The two-factor ANOVA including the Hamilton hound gives equivalent results as when it is excluded (*F*_9,18_ = 26.74, P<<0.001 and *F*_2,18_ = 2.50, P = 0.11, for breeds and time points, respectively). The observation of differences in inbreeding levels between breeds and between time points is supported also when quantifying inbreeding in terms of *MK* or as average *N*_e_ over generations (data not shown).

When analyzing each breed separately there is a weak but consistent increase of inbreeding over time within all breeds (S1 Fig) with linear regression coefficients (*b*) in the range 0.001–0.004, and all P-values <<0.001. These observations are consistent for the Gotland hound, Swedish white elkhound and the Danish-Swedish farmdog both with and without the 1980 data.

### Loss of founder allele variation–differences among breeds and over time

Loss of genetic variation measured as number of remaining founder alleles over the monitored time period is extensive. In 2012 the proportion of remaining founder alleles ranges from 0.13 (Hamilton hound) to 0.49 (Danish-Swedish farmdog) with an average over breeds of 0.29. This suggests that on average 70 percent of the genetic variation measured by this pedigree based parameter has been lost over a c. 5 decade period representing around 8 generations (average number of generations per breed in 2012; cf. Table 2).

Similar results are observed when retention of founder alleles is quantified as number of founder genome equivalents (*fge*). The range of *fge* among live dogs in 2012 is 3.3–11.5 with the Gotland hound representing the lowest values and the Swedish white elkhound the largest (Table 2). The interpretation of this result is that the 2012 genepools of these breeds include genetic variation of the magnitude of 3.3–11.5 unrelated founders [22]. The averages *fge* = 7.1 over all breeds indicates that on average the genetic variation of separate breeds could be represented by 7 unrelated individuals.

**Fig 3:**
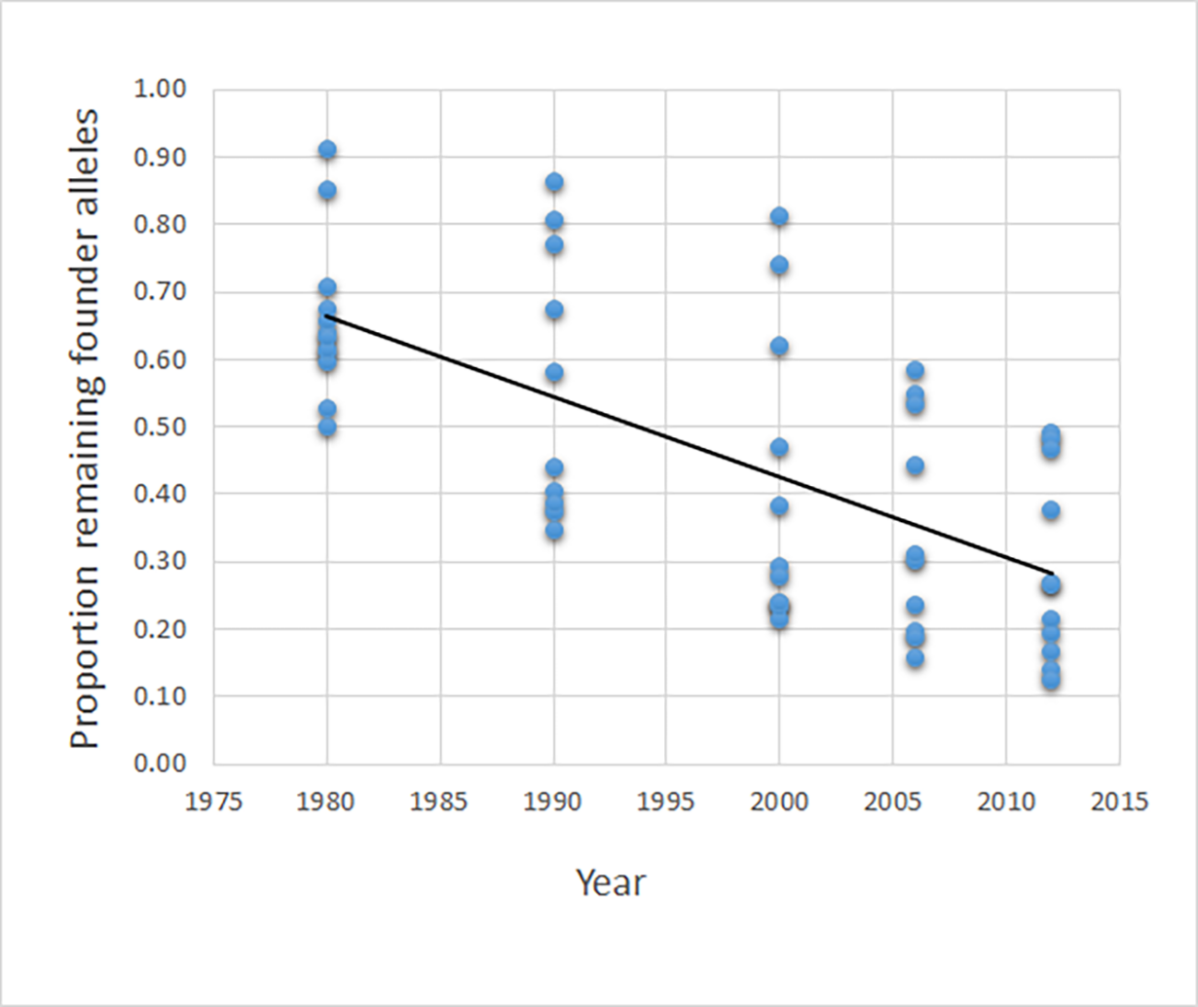
Proportion of founder alleles that remain in each of 12 traditional Swedish dog breeds at five points in time over a 32 year period monitored in this study. Linear regression gives *b* = -0.012, *r*^2^ = 0.42, P<<0.001 and the regression line is shown.

There is a highly significant negative trend over time in the proportion of remaining founder alleles, but the variation among breeds is quite large (*b* = -0.012, *r*^2^ = 0.42, P<<0.001; Fig 3). Two-factor ANOVA without replication for 1980–2012 (including the nine breeds having results for all five time points) gives significance for both between points in time (*F*_4,32_ = 16.65, P<<0.001) and between breeds (*F*_8,32_ = 8.69, P<<0.001). Similar variation in proportion of remaining founder alleles exists over the last part of the period (2000–2012; *F*_9,18_ = 30.02, P<0.0001 and *F*_2,18_ = 13.01, P<0.001, for breeds and time points, respectively), and is observed also when retention of genetic variation is measured as the number of *fge* per founder. Two-factor ANOVAs without replication for *fge* over 1980–2012 gives between breeds *F*_8,32_ = 7.39 (P<0.0001) and between points in time *F*_4,32_ = 6.69 (P<0.001). The same test gives equivalent results for *fge* over 2000–2012 (among breeds *F*_9,18_ = 98.4 and 115.9, between time point *F*_2,18_ = 10.9 and 13.9, all P<0.001).

### Effects of assumption on longevity

We had to make assumptions regarding longevity because the database of the Swedish Kennel Club does not include information on when individual animals die. We have assumed that all dogs of all breeds live for 12 years and then die. Clearly, this is not accurate and it is important to consider if and how this assumption affects the generality of our results. The pedigrees themselves will, of course, not be affected by assumptions on longevity, but assessments of inbreeding and loss of founder alleles at particular points in time might be. Similarly, generation length and number founders of living animals at various time points could also be affected and, thus, quantification of average *N*_e_ over generations.

We performed additional analyses where the assumption regarding lifespan was set to 6 years and the results are very similar as when assuming a lifespan of 12 years (Table 3). The trend of an increase in inbreeding over time persists also with a 6 year lifespan (linear regression gives *b* = 0.001, *r*^2^ = 0.18, P<0.001; cf Fig 2 for the relationship with when lifespan is 12 years), as does the trend of a decreasing proportion of founder alleles remaining over time (linear regression gives *b* = -0.010, *r*^2^ = 0.35, P<0.00001; cf Fig 3 for the relationship with when lifespan is 12 years). Similarly, average *N*_*e*_ over generations is very similar regardless of lifespan. However, the parameters current *N*_*e*_ and current *N*_*e*_/*N* are strongly affected (Table 3).

**Table 3:** Average summary statistics from pedigree data over breeds obtained when assuming a lifespan of 6 vs. 12 years.

	Lifespan (years)	Year				
		1980	1990	2000	2006	2012
**Number of breeds**	6	12	12	12	12	12
**possible to analyze**	12	12	10	11	10	11
***A*verage inbreeding**	6	0.036	0.050	0.065	0.068	0.067
**coefficient (*F*)**	12	0.033	0.045	0.060	0.065	0.066
**Mean kinship (*MK*)**	6	0.066	0.059	0.076	0.080	0.083
** **	12	0.063	0.055	0.068	0.076	0.080
**Proportion of founder alleles remaining**^**1**^	6	0.544	0.450	0.314	0.273	0.245
** **	12	0.661	0.565	0.424	0.350	0.291
***fge*/founder**^**2**^	6	0.237	0.157	0.115	0.095	0.089
** **	12	0.237	0.178	0.123	0.114	0.094
***N***_***e***_^**3**^	6	45.2	48.5	52.3	55.9	61.2
** **	12	46.7	44.1	51.0	52.7	59.1
**Number of generations (*G*)**	6	3.5	5.1	6.9	7.9	8.9
** **	12	3.3	4.3	6.1	7.0	8.2
**Current *N***_***e***_^**4**^	6	201.0	164.8	111.2	104.4	99.6
** **	12	799.5	330.3	368.9	223.0	282.4
**Current *N***_***e***_***/N***^**5**^	6	0.073	0.071	0.052	0.052	0.052
	12	0.180	0.129	0.113	0.114	0.116

^**1**^The proportion of the initial number of founder alleles (defined as twice the number of founders) that is retained in one or more copies among living dogs.

^2^The number of founder genome equivalents divided by the number of founders that are genetically represented in living animals.

^3^Average genetically effective size over generations.

^4^*N*_e_ = 4*N*_m_*N*_f_/(*N*_m_+*N*_f_), where *N*_m_ = number of live males that have reproduced and *N*_f_ = number of living females that have reproduced.

^5^Current *N*_*e*_ divided by the current census size (*N*) i.e., the number of living animals when assuming a maximum life span of either 6 or 12 years.

### Effects of removing individuals carrying deleterious alleles

For several of the Swedish national dog breeds, hereditary defects occur and the SKC has set up so-called disease control programs for some of these disorders (cf. Table 1). Such disease control programs generally imply that animals should have a diagnosis with respect to the defect prior to breeding. Diagnosis might imply veterinary examination (necessary for diagnosing e.g., hip dysplasia that occur in many of the breeds) and/or genotypic screening (available for e.g., the progressive retinal atrophy that is caused by a single recessive allele which occurs in the Swedish lapphund and the Norrbottenspitz).

The Swedish lapphund exhibits prcd-PRA—an eye disease that results in blindness. The defect is caused by a single autosomal, recessive allele [27]. The SKC´s control program allows heterozygous carriers (type B), but not affected dogs (type C), to be included in breeding. SKC requires a DNA test to determine the genotype of individual dogs (http://www.skk.se).

We analyzed the effect on the conservation genetic status of the Swedish lapphund population of removing not only type C dogs but also type B dogs (heterozygous carriers) from the population. We proceeded as follows: from the population of animals classified as alive (using the assumption 12 years as the maximum age) as of December 31, 2012 we removed type B and C dogs. This resulted in removal of 34 percent of the population. We then assessed the variables studied (population average *F*, *MK*, *fge*). The removal did not affect average inbreeding among remaining animals (*F* = 0.079 versus 0.081 with and without removal, respectively), and neither was mean kinship (*MK* = 0.085 versus 0.084) or *fge* (5.95 versus 5.93) among remaining animals affected.

With respect to the Norrbottenspitz only few animals suffer from PRA; up until 2009 only 10 animals had shown the defect, and in the population of 2012 only 15 heterozygous carriers had been confirmed. We did not perform an analysis of the effects of removing these animals.

We also analyzed the Swedish lapphund pedigree in regard to an additional hereditary defect that occurs in the population—hip dysplasia. This defect is also considered hereditary [28], and we obtained similar results for this character as for prcd-PRA. Excluding dogs affected with hip dysplasia categories C (mild dysplasia), D (moderate dysplasia), or E (high grad of dysplasia) did not affect average level of inbreeding (*F* = 0.083 versus 0.082 with and without removal, respectively), mean kinship (*MK* = 0.089 versus 0.084), or *fge* (5.63 versus 5.67).

The same pattern regarding conservation genetic effects of removing dogs affected with hip dysplasia (HD) were observed in the Danish-Swedish farmdog and Swedish white elkhound populations. Excluding HD affected dogs (classified as C, D or E) for Danish-Swedish farmdogs did not affect average inbreeding among live animals in 2012 (*F* = 0.040 versus 0.041 with and without removal, respectively), mean kinship (*MK* = 0.044 in both cases) or *fge* (11.31 versus 11.32). For the Swedish white elkhound average inbreeding with and without removal were *F* = 0.018 in both cases, mean kinship was *MK* = 0.043 versus 0.044 with and without removal of HD affected dogs, and *fge* was 11.43 versus 11.49.

## Discussion

We have monitored inbreeding levels and retention of intra-breed variation using pedigree data from 12 traditional Swedish dog breeds, ten of which have been identified as of national conservation concern [9], over a 32 year period, 1980–2012, and our main observations are:

1. Inbreeding (*F*) increases over this period and in 2012 averages for separate breeds are in the range $\bar{F}$ = 0.02–0.10.
2. Loss of founder alleles is extensive; the proportion of founder alleles that have been lost in different breeds ranges from c. 50–90 percent.
3. Founder allelic variation of 2012 gene pools was equivalent to only 3–20 founding animals, which reflects a loss of over 80 percent of founder variation measured as founder genome equivalents.
4. Effective population size (*N*_e_) over generations is slightly above the critical level of 50 [29] for the majority of breeds, and for two breeds (Gotland hound, Hällefors elkhound) *N*_e_ is below this level.
5. Rates of inbreeding and loss of variation is unnecessarily extensive considering the census sizes of these breeds. This is indicated by the ratio between current *N*_*e*_*−*reflecting number of males and females used in breeding corrected for uneven sex ratio–and number of live animals which is often less than 20 percent and often only around 10 percent (Table 2).

Our observations are in line with previous findings; about 90 percent loss of pedigree measured genetic variation during the past few decades has been reported in nine dog breeds in France [14], three scent hound breeds in central Europe [15], ten dog populations bred in the United Kingdom [16], and 26 dog populations bred in Sweden [17]. Clearly, present day dog breeding appears to be associated with a rapid loss of genetic variation. Our present results indicate that this is true even for breeds that have been identified as of specific conservation value, and where breeding goals explicitly include maintaining genetic variation.

These observations are worrying since reduced genetic variation and inbreeding are generally associated with loss of adaptive potential and reduced options for effective selection [30]. We note that this rapid genetic diversity loss is paralleled to increasing needs of dogs for a number of different purposes in modern society [10, 11, 12, 13]. Similarly, elevated health problems in dogs are frequently associated with their genetic background [31]. While the importance of conserving genetic resources of domestic species are recognized in international policy; a specific biodiversity target of the CBD Strategic Plan 2011–2020—Aichi target 13 –is even directed towards such resources, implementation lags behind.

### Genomic studies needed

Clearly, our present study and other studies reporting rapid increase of inbreeding and loss of pedigree-measured genetic variation need to be supplemented with genomic studies in order to find out the extent to which the trends indicated by the breeding records are reflected over the genomes of these breeds. Considerable contemporary research is focused on various aspects of genomic characterization of domestic dog breeds [32, 33, 34]. Recent studies report contrasting results with respect to correlation between pedigree inbreeding coefficients (FPed) and genomic runs of homozygosity (ROH). Some researchers find good correlations between FPed and ROH in domestic dogs [35] as well as in wolves [36], whereas others report that such correlations are poor [37, 38]. Similarly, only moderate correlations between FPed and ROH have been found in e.g. cattle [39, 40]. Thus, pedigree based estimates of inbreeding levels and amount of genetic diversity may not provide a complete picture of genomic levels of genetic diversity. Also, since the pedigree data used here only dates back to around the mid 1900s we do not have a complete picture of the history of these breeds which are typically much older [18], and genomic data would provide most valuable information in this respect.

Genomic marker-based measures have been suggested to outperform pedigrees [41, 42], but until such tools become widely available, including to breeders, we think that pedigree analysis provides a cost-effective opportunity for planning breeding and monitoring rates of recent inbreeding and loss of variation. Such pedigree based conservation breeding approaches focus on minimizing mean kinship (*MK*) and has long been applied within the context of maintaining endangered wild animal species in zoos [25]. A recent study reported that such conservation breeding protocols also effectively retained genomic diversity [43].

### Findings in relation to conservation breeding goals

With respect to the situation for the 12 breeds investigated in the present study current breeding goals are not well met with respect to retention of pedigree measured genetic variation. An exception is the Gotland hound which is numerically very small but where several quantities indicate that breeding has been carried out well from a conservation perspective. The ratio of remaining founder genome equivalents to the number of founders is almost 30 percent for this breed (*fge*/number of founders = 3.31/12 = 0.276) which is much higher than for any of the other breeds. Similarly, current *N*_*e*_/*N* is highest (0.16) for the Gotland hound and the proportion of remaining founder alleles is around 50 percent which is the highest retention observed (also reached for the Danish Swedish Farmdog and the Swedish white elkhound).

The Swedish Kennel Club recommends that mating resulting in *F*>0.0625 should be avoided (SKC website assessed in April 2018: http://www.skk.se/kopahund/att-kopa-hund/hundars-halsa/inavelsgrad/ [In Swedish]). This recommendation is not met for 8 of the 12 breeds where average *F* of the living population exceeds 0.0625 at one or more points in time, and the upper range is practically always well above *F* = 0.0625 (Table 2). Similarly, the SKC recommends that increase of inbreeding should not exceed 0.5 percent per generation (SKC website assessed in April 2018: http://www.skk.se/kopahund/att-kopa-hund/hundars-halsa/inavelsgrad/[In Swedish]) implying a per generation *N*_e_ ≥100. This criterion is violated for all breeds over the full study period.

It is interesting to note that for none of the breeds, with the exception of the Gotland hound, the rates of inbreeding and the rate of loss of founder alleles seem to be due to small population size. Rather, it appears that the reason for rapid loss of variation and increasing inbreeding includes the use of only an unnecessarily small proportion of animals in breeding. This is indicated by the ratios of current *N*_e_/*N* (Tables 2 and 3); in 2012 this ratio varied between 0.06–0.16 (average 0.12).

### Threat classification according to FAO criteria

The FAO criteria for threat classification [8] have been used by the Swedish Board of Agriculture to classify all Swedish native domestic animal breeds including the ten native dog breeds identified as of conservation concern [24]. Six of the breeds were classified as Critical-maintained and four as Endangered-maintained (Table 1). The FAO criteria for domestic breeds might not be as well known to the general biologist/geneticist as the IUCN Red List categories used for wild animal species [44] (www.iucnredlist.org), and we therefore describe them briefly here. The FAO categories include Extinct, Critical, Critical-maintained, Endangered, Endangered-maintained, Not at risk, and Unknown. The category Extinct is met when the breed no longer exists or when only individuals of one sex remain. Critical is met if *i*) the total number of breeding females is less than or equal to 100 or *ii*) the total number of breeding males is less than or equal to five; or *iii*) if the overall population size is less than or equal to 120 and decreasing and the percentage of females being bred to males of the same breed is below 80 percent.

The Endangered category implies that *i*) the total number of breeding females is greater than 100 and less than or equal to 1,000 or *ii*) the total number of breeding males is less than or equal to 20 and greater than five; or *iii*) if the overall population size is greater than 80 and less than 100 and increasing and the percentage of females being bred to males of the same breed is above 80 percent; or *iv*) if the overall population size is greater than 1,000 and less than or equal to 1200 and decreasing and the percentage of females being bred to males of the same breed is below 80 percent. The classification as Critical-maintained or Endangered-maintained implies that the category Critical or Endangered is met but that active conservation programs are in place. Category Not at risk refers to when none of the threatened criteria are met, e.g. the total number of breeding females and males are greater than 1,000 and 20, respectively, or if the population size is greater than 1,200 and the overall population size is increasing.

There are obvious shortcomings of the FAO classification system (as of any such system). For instance, no direct genetic parameters are included [45]. However, it might still be valuable to apply this classification system to get a general picture of the status of various domestic breeds that can be monitored over time in addition to various conservation genetic parameters. The first and only classification of Swedish dog breeds was presented by the Swedish Board of Agriculture in 2007 [24], and there is a need to update these classifications.

### Effects of selective breeding on genetic diversity retention

There is an ongoing discussion of potential conservation genetic effects of imposing selective breeding aiming at removing hereditary disorders in these breeds. Animals with a diagnosed defect can, in many cases, still be used in breeding. For example, dogs with diagnosed hip dysplasia can be used in breeding although the SKC and breed clubs often recommend that they are not. Similarly, animals that are known carriers of the allele that causes progressive retinal atrophy (PRA) can be used in breeding if they are mated to documented (through genotypic screening) non-carriers. Dogs homozygous for the PRA-allele cannot be used in breeding. One reason for allowing the use of carriers in breeding is because if those animals are selected against this would result in extensive loss of genetic variation.

Previous studies on wild animals bred in captivity for conservation purposes have shown that evaluation of effects of selective removal of animals carrying single deleterious alleles must be carried out on a case by case basis. For instance, removing high probability carriers of a single allele causing blindness in a captive wolf population did not affect retention of overall founder genetic variation. In contrast, removal of high probability carriers of an allele causing albinism in a captive brown bear population could not be done without substantial loss of genetic variation [46].

Our present results indicate that selective removal of affected animals can often be carried out without further loss of founder genetic variation. For the present breeds this seems to be the case for hip dysplasia in the Swedish lapphund, the Swedish white elkhound, and the Danish-Swedish farmdog and also for progressive retinal atrophy in Swedish lapphund._._

### Concluding recommendations

In order to reduce the rate of loss of genetic variation and increase of inbreeding that has been documented in many dog breeds we recommend that conservation genetic criteria such as *MK* rankings are included when selecting animals for breeding. Further, a larger portion of existing dogs should be used in breeding. For the breeds of the present study current census sizes imply that future increase of inbreeding and loss of genetic variation has the potential to be miniscule if more dogs are used in breeding and extreme variation in offspring production between separate dogs are avoided. We note that both software applied here (PMx and mPed) are freely available and genetic monitoring using pedigree data is a cost effective method to improve breeding from a conservation perspective. It is important, however, that supplementary genomic studies are conducted for these and other dog breeds in order to find out, e.g., how well the pedigree measurements reflect genomic inbreeding and levels of genetic variation.

Our present analysis does not cover much of the period 2010–2020 during which the Strategic Plan of the Convention of Biological Diversity applies and where a specific Target referring to genetic diversity of domestic species applies (Aichi Target 13; http://www.cbd.int/sp/). Thus, we urge for continued monitoring of these populations including the use of genomic tools. We also stress the importance of documenting dates of death of separate dogs in the SKC database to provide an opportunity of obtaining as correct a picture as possible with respect to the conservation genetic situation.

## Supporting information

S1 Fig Inbreeding levels (*F*) over the monitored time period 1980–2012 for each of the 12 native Swedish dog breeds monitored in this study. (PDF)

## References

[1] Taberlet P, Coissac E, Pansu J, Pompanon F. Conservation genetics of cattle, sheep, and goats. C R Biol. 2011; 334: 247–254. 10.1016/j.crvi.2010.12.007 21377620

[2] Baumung R, Simianer H, Hoffman I. Genetic diversity studies in farm animals–a survey. J Anim Breed Genet. 2004; 121: 361–373. 10.1111/j.1439-0388.2004.00479.x

[3] Martyniuk E, Pilling D, Scherf B. Indicators: Do we have effective trends in genetic diversity of domesticated animals? Anim Genet Resour. 2010; 47: 31–43. 10.1017/S2078633610001013

[4] Yang H. Livestock development in China: animal production, consumption and genetic resources. J Anim Breed Genet. 2013; 130: 249–251. 10.1111/jbg.12045 23855626

[5] FAO. Global Plan of Action for Animal Genetic Resources and the Interlaken Declaration, adopted by the International Technical Conference on Animal Genetic Resources for Food and Agriculture Interlaken, Switzerland, 3–7 September 2007 Commission on Genetic Resources for Food and Agriculture of the Food and Agriculture Organization of the United Nations, FAO, Rome, Italy Available from: www.fao.org.

[6] Leroy G. Genetic diversity, inbreeding and breeding practices in dogs: Results from pedigree analyses. Vet J. 2011; 189: 177–182. 10.1016/j.tvjl.2011.06.016 21737321

[7] Stephens TD, Splan RK. Population history and genetic variability of the American Shire horse. Anim Genet Resour. 2013; 52: 31–38. 10.1017/S2078633613000052

[8] Scherf BD. World watch list for domestic animal diversity, 3rd edition Food and Agriculture Organization of the United Nations, Rome, Italy, 10 2000 Available from: www.fao.org.

[9] Swedish Board of Agriculture. Action plan for the long-term sustainable management of Swedish domestic animal genetic resources 2010–2020. 2009. [In Swedish]. Available from: www.jordbruksverket.se.

[10] Browne C, Stafford K, Fordham R. The use of scent-detection dogs. Irish Vet J. 2006; 59: 97–104.

[11] Horvath G, af Klinteberg Järverud G, Järverud S, Horváth I. Human ovarian carcinomas detected by specific odor. Integr Cancer Ther. 2008; 7: 76–80. 10.1177/1534735408319058 18505901

[12] Lindblad-Toh K, Wade CM, Mikkelsen TS, Karlsson EK, Jaffe DB, Kamal M, et al Genome sequence, comparative analysis and haplotype structure of the domestic dog. Nature. 2005; 438: 803–819. 10.1038/nature04338 16341006

[13] Wells DL. Domestic dogs and human health: An overview. Br J Health Psychol. 2007; 12: 145–156. 10.1348/135910706X103284 17288671

[14] Leroy G, Rognon X, Varlet A, Joffrin C, Verrier E. Genetic variability in French dog breeds assessed by pedigree data. J Anim Breed Genet. 2006; 123: 1–9. 10.1111/j.1439-0388.2006.00565.x 16420259

[15] Voges S, Distl O. Inbreeding trends and pedigree analysis of Bavarian mountain hounds, Hanoverian hounds and Tyrolean hounds. J Anim Breed Genet. 2009; 126: 357–365. 10.1111/j.1439-0388.2009.00800.x 19765162

[16] Calboli FCF, Sampson J, Fretwell N, Balding DJ. Population structure and inbreeding from pedigree analysis of purebred dogs. Genetics. 2008; 179: 593–601. 10.1534/genetics.107.084954 18493074 PMC2390636

[17] Jansson M, Laikre L. Recent breeding history of dog breeds in Sweden: modest rates of inbreeding, extensive loss of genetic diversity, and lack of correlation between inbreeding and health. J Anim Breed Genet. 2014; 131: 153–162. 10.1111/jbg.12060 24289536 PMC4166703

[18] Lindholm Å. Swedish native breeds: a cultural heritage The Swedish Kennel Club 2007.

[19] Willes R. Swedish breeds of dogs The Swedish Kennel Club 2008 www.skk.se/globalassets/dokument/hundrasguiden/svenska-raser.pdf

[20] Unsgård J. Native dog breeds of Norway The Norwegian Kennel Club 2015 http://www.nkkbutikken.no/butikk/boker/forhandssalg-norske-raser-bok/

[21] Dog Care Professionals. The Icelandic Sheepdog. A Complete and Comprehensive Owners Guide to: Buying, Owning, Health, Grooming, Training, Obedience, Understanding and Caring for Your Icelandic Sheepdog DestinyGate Publishing & Distribution; 2016.

[22] Lacy RC. Analysis of founder representation in pedigrees–Founder equivalents and founder genome equivalents. Zoo Biol. 1989; 8: 111–123. 10.1002/zoo.1430080203

[23] Lacy RC. Clarification of genetic terms and their use in the management of captive populations. Zoo Biol. 1995; 14: 565–578. 10.1002/zoo.1430140609

[24] Lannek J. Goals for domestic animal genetic resources during 2010–2020. Report, Swedish Board of Agriculture, Jönköping, Sweden. 2007. [In Swedish]. Available from: www.jordbruksverket.se

[25] Lacy RC, Ballou JD, Pollak JP. PMx: Software package for demographic and genetic analysis and management of pedigreed populations. Methods Ecol Evol. 2012; 3: 433–437. 10.1111/j.2041-210X.2011.00148.x

[26] Jansson M, Ståhl I, Laikre L. mPed: a computer program for converting pedigree data to a format used by the PMx-software for conservation genetic analysis. Conserv Genet Resour. 2013; 5: 651–653. 10.1007/s12686-013-9874-z

[27] Mellersh C. DNA testing and domestic dogs. Mamm Genome. 2012; 23: 109–123. 10.1007/s00335-011-9365-z 22071879 PMC3275738

[28] Bartolomé N, Segarra S, Artieda M, Francino O, Sánchez E, Szczypiorska M, et al A genetic predictive model for canine hip dysplasia: integration of genome wide association study (GWAS) and candidate gene approaches. PLoS One. 2015; 10(4): e0122558 10.1371/journal.pone.0122558 25874693 PMC4395148

[29] Frankel OH, Soulé ME. Conservation and Evolution Cambridge University Press; 1981.

[30] Allendorf FW, Luikart GH, Aitken SN. Conservation and the Genetics of Populations, 2nd Edition Wiley-Blackwell; 2012.

[31] Donner J, Kaukonen M, Anderson H, Möller F, Kyöstilä K, Sankari S, et al Genetic panel screening of nearly 100 mutations reveals new insights into the breed distribution of risk variants for canine hereditary disorders. PLoS One. 2016; 11(8): e0161005 10.1371/journal.pone.0161005 27525650 PMC4985128

[32] Parker HG. Genomic analyses of modern dog breeds. Mamm Genome. 2012; 23: 19–27. 10.1007/s00335-011-9387-6 22231497 PMC3559126

[33] Parker HG, Dreger DL, Rimbault M, Davis BW, Mullen AB, Carpintero-Ramirez G, et al Genomic analyses reveal the influence of geographic origin, migration, and hybridization on modern dog breed development. Cell Rep. 2017; 19: 697–708. 10.1016/j.celrep.2017.03.079 28445722 PMC5492993

[34] Serres-Armero A, Povolotskaya IS, Quilez J, Ramirez O, Santpere G, Kuderna LKF, et al Similar genomic proportions of copy number variation within gray wolves and modern dog breeds inferred from whole genome sequencing. BMC Genomics. 2017; 18:977 10.1186/s12864-017-4318-x 29258433 PMC5735816

[35] Wiener P, Sánchez-Molano E, Clements DN, Woolliams JA, Haskell MJ, Blott SC. Genomic data illuminates demography, genetic structure and selection of a popular dog breed. BMC Genomics. 2017; 18:609 10.1186/s12864-017-3933-x 28806925 PMC5557481

[36] Kardos M, Åkesson M, Fountain T, Flagstad Ø, Liberg O, Olason P, et al Genomic consequences of intensive inbreeding in an isolated wolf population. Nat Ecol Evol. 2017; 2: 124–131. 10.1038/s41559-017-0375-4 29158554

[37] Wade CM. Inbreeding and genetic diversity in dogs: Results from DNA analysis. The Vet J. 2011; 189:183–188. 10.1016/j.tvjl.2011.06.017 21745753

[38] Deger DL, Rimbault M, Davis BW, Bhatnagar A, Parker HG, Ostrander EA. Whole-genome sequence, SNP chips and pedigree structure: building demographic profiles in domestic dog breeds to optimize genetic-trait mapping. Dis Model Mech. 2016; 9: 1445–1460. 10.1242/dmm.027037 27874836 PMC5200897

[39] Marras G, Gaspa G, Sorbolini S, Dimauro C, Ajmone-Marsan P, Valentini A, et al Analysis of runs of homozygosity and their relationship with inbreeding in five cattle breeds farmed in Italy. Anim Genet. 2015; 46:110–121. 10.1111/age.12259 25530322

[40] Kim E-S, Sonstegard TS, Van Tassell CP, Wiggans G, Rothschild MF. The relationship between runs of homozygosity and inbreeding in Jersey cattle under selection. PLoS ONE. 2015; 10(7): e0129967 10.1371/journal.pone.0129967 26154171 PMC4496098

[41] Kardos M, Luikart G, Allendorf FW. Measuring individual inbreeding in the age of genomics: marker-based measures are better than pedigrees. Heredity 2015; 115:63–72. 10.1038/hdy.2015.17 26059970 PMC4815495

[42] Speed D, Balding DJ. Relatedness in the post-genomic era: is it still useful? Nat Rev Genet. 2015; 16: 33–44. 10.1038/nrg3821 25404112

[43] Willoughby JR, Ivy JA, Lacy RC, Doyle JM, DeWoody JA. Inbreeding and selection shape genomic diversity in captive populations: Implications for the conservation of endangered species. PLoS ONE. 2017; 12(4): e0175996 10.1371/journal.pone.0175996 28423000 PMC5396937

[44] Mace GM, Collar NJ, Gaston KJ, Hilton-Taylor C, Akçakaya HR, Leader-Williams N, et al Quantification of extinction risk: IUCN’s System for Classifying Threatened Species. Conserv Biol. 2008; 22: 1424–1442. 10.1111/j.1523-1739.2008.01044.x 18847444

[45] Laikre L. Genetic diversity is overlooked in international conservation policy implementation. Conserv Genet. 2010; 11: 349–354. 10.1007/s10592-009-0037-4

[46] Laikre L. Conservation genetics of Nordic carnivores: lessons from zoos. Hereditas. 1999; 130: 203–216. 10.1111/j.1601-5223.1999.00203.x 10509137

