# Supplementary material for: Pedigree data indicate rapid inbreeding and loss of genetic diversity within populations of native, traditional dog breeds of conservation concern
Source: PLoS One. 2018 Sep 12;13(9):e0202849. doi: 10.1371/journal.pone.0202849 (PMC6135370; doi:10.1371/journal.pone.0202849)
Supplement: S1 Fig — (PDF) [file pone.0202849.s001.pdf]

Supporting information

Rapid loss of genetic variation in native, traditional Swedish dog breeds of conservation concern

By Mija Jansson and Linda Laikre

Figure S1. Inbreeding levels (*F*) over time for separate Swedish dog breeds. The following quantities are shown for each breed and point in time: the median (solid, thick black line), the lower and upper quartiles (lower and upper box limits, respectively), and the smallest and largest observations (lower and upper horizontal lines, respectively). Values regarded as outliers are indicated by open circles. For the Danish-Swedish farmdog, Gotland hound, Hällefors hound, and the Swedish white elkhound, there is not enough variation among dogs alive at separate points in time to provide quartiles; smallest and largest values coincide with outliers and are indicated by circles (cf. Table 2).

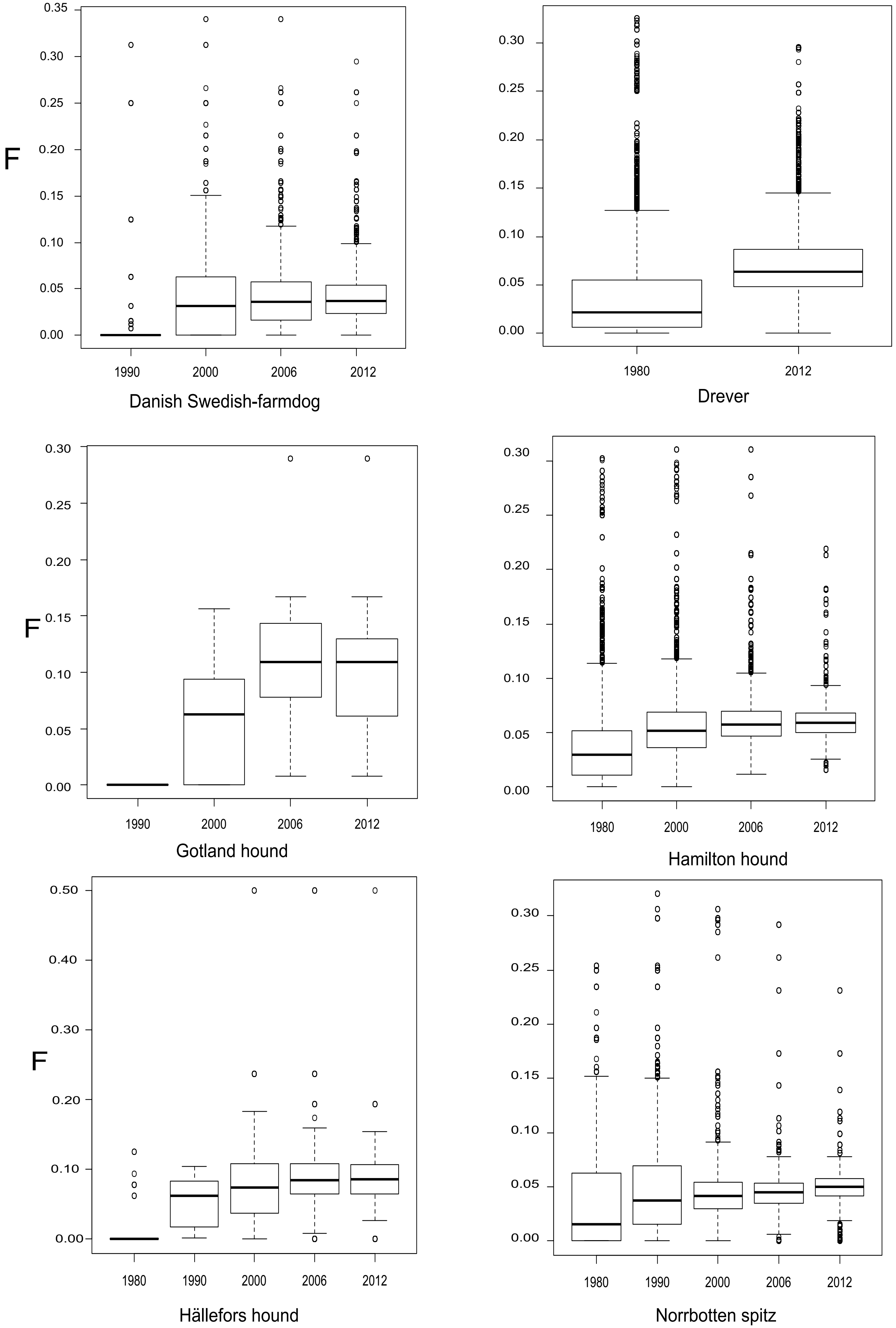
F
0.00
0.05
0.10
0.15
0.20
0.25
0.30
0.35
1990
2000
2006
2012
Danish Swedish-farmdog
0.00
0.05
0.10
0.15
0.20
0.25
0.30
1980
2012
Drever
F
0.00
0.05
0.10
0.15
0.20
0.25
0.30
1990
2000
2006
2012
Gotland hound
0.00
0.05
0.10
0.15
0.20
0.25
0.30
1980
2000
2006
2012
Hamilton hound
F
0.00
0.10
0.20
0.30
0.40
0.50
1980
1990
2000
2006
2012
Hällefors hound
0.00
0.05
0.10
0.15
0.20
0.25
0.30
1980
1990
2000
2006
2012
Norrbotten spitz

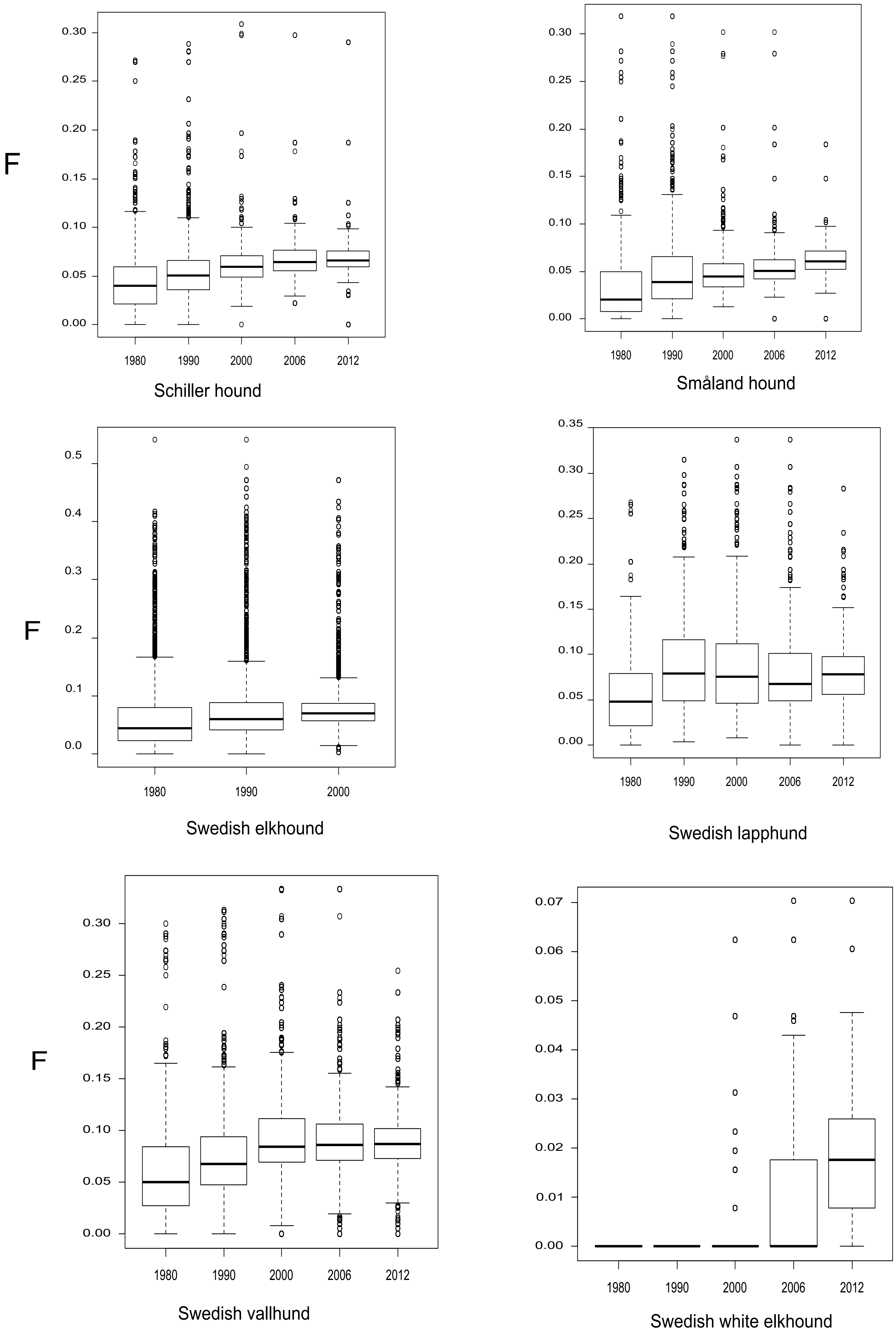
F
0.00
0.05
0.10
0.15
0.20
0.25
0.30
1980
1990
2000
2006
2012
Schiller hound
Småland hound
0.0
0.1
0.2
0.3
0.4
0.5
Swedish elkhound
0.35
Swedish lapphund
Swedish vallhund
0.01
0.02
0.03
0.04
0.05
0.06
0.07
Swedish white elkhound
